# Supplementary material for: Effect of intentional restriction of venous return on tissue oxygenation in a porcine model of acute limb ischemia
Source: PLoS One. 2020 Dec 14;15(12):e0243033. doi: 10.1371/journal.pone.0243033 (PMC7735909; doi:10.1371/journal.pone.0243033)
Supplement: S1 Table — (PDF) [file pone.0243033.s001.pdf]

Table 1. Blood sampling of the SFA and CIV at different moments (From T0 to T7).

SFA (percent)		CIV (percent)	
T0	100	T0	85.57 ± 1.72
T1	98.43 ± 1.27	T1	75.43 ± 8.44
T2	98.29 ± 1.11	T2	71.71 ± 5.93
T3	97.57 ± 1.51	T3	72.57 ± 6.35
T4	97.71 ± 1.97	T4	71.86 ± 7.58
T5	98.29 ± 2.13	T5	74.14 ± 9.56
T6	98.29 ± 1.60	T6	79.29 ± 4.82
T7	98.57 ± 0.97	T7	79.71 ± 4.78

Values are n (%) or mean ± SD, as appropriate. Abbreviations: SFA, supercifical femoral artery; CIV, common iliac vein.
