# Supplementary material for: Effect of intentional restriction of venous return on tissue oxygenation in a porcine model of acute limb ischemia
Source: PLoS One. 2020 Dec 14;15(12):e0243033. doi: 10.1371/journal.pone.0243033 (PMC7735909; doi:10.1371/journal.pone.0243033)
Supplement: S2 Table — (PDF) [file pone.0243033.s002.pdf]

Table 2. Comparision of the venous oxygen saturations between the experimental limbs with arterial embolization and the control limbs.

	Experimental limb	Control limb	p-value
T5	74.14 ± 9.56	57.50 ± 7.77	0.061
T6	79.28 ± 4.82	59.00 ± 2.82	< 0.001
T7	79.71 ± 4.78	60.00 ± 4.24	< 0.001

Values are n (%) or mean ± SD, as appropriate.
