# Supplementary material for: Effect of intentional restriction of venous return on tissue oxygenation in a porcine model of acute limb ischemia
Source: PLoS One. 2020 Dec 14;15(12):e0243033. doi: 10.1371/journal.pone.0243033 (PMC7735909; doi:10.1371/journal.pone.0243033)
Supplement: Attachment — Submitted filename: Review.docx [file pone.0243033.s004.docx]

The authors present a single institution study looking at the effect of intentional restriction of venous return on tissue oxygenation in a porcine model of acute limb ischemia and investigate whether partial venous occlusion in the common iliac vein (CIV) causes a further increase of venous oxygenation in a porcine model of acute hindlimb ischemia.

. Just unter 10 porcines were analyzed. While this is interesting work, I have some questions and suggestions for revisions:

I think the number of the study is to small to draw conculsion, the authors should soften their statements

In Discussion part line 15 “..An expectation for this may be that the oxygen consumption in the limb tissue is increased..” do you mean expectation or explanation . I think you mean explanation.

The pathophysiological reasons of venous occlusion and increased oxygen saturation is not becoming clear. Please discuss it better. How do you explain this inthe peripheral system?

We know in the peripheral system a venous congestion results in trophic skin disorder and venus ulcera and is contraindicated for wound healing.

How do you measure the saturation, this should be clarified.

Page 3 line 21-23 : The authors try to transferr the pathophysiological process in the heart to the perpheral vessels. I think this need to be discussed much deeper.

Arterial occlusion combined with venous occlusion results in the peripheral system to the situation phlegmasia coerlia dolens, which is a life threating condition. Who was lactat, do you measure any labor parameters.
