# Supplementary material for: Effect of intentional restriction of venous return on tissue oxygenation in a porcine model of acute limb ischemia
Source: PLoS One. 2020 Dec 14;15(12):e0243033. doi: 10.1371/journal.pone.0243033 (PMC7735909; doi:10.1371/journal.pone.0243033)
Supplement: Attachment — Submitted filename: Response to Reviewers (CIV occlusion study).doc [file pone.0243033.s005.doc]

**Journal Requirements:**

When submitting your revision, we need you to address these additional requirements.

1. Please ensure that your manuscript meets PLOS ONE's style requirements, including those for file naming. The PLOS ONE style templates can be found at

https://journals.plos.org/plosone/s/file?id=wjVg/PLOSOne_formatting_sample_main_body.pdf and

https://journals.plos.org/plosone/s/file?id=ba62/PLOSOne_formatting_sample_title_authors_affiliations.pdf

2.At this time, we request that you please report additional details in your Methods section regarding animal care, as per our editorial guidelines:

(1) Please state the source of the pigs used in the study

A sentence was added in the method section as below :

The animals used in this study were miniature pigs originated from Korean Jeju island native (Cronex Co., Ltd., Osong, Republic of Korea).

(2) Please provide details of animal welfare (e.g., shelter, food, water, environmental enrichment) : Sentences were added in the method section as below :

The animals were housed individually following standard laboratory conditions (temperature: 19-25 ˚C; humidity: 30-70%; ventilation: 10-15 per hour; light: 150-300 Lux; light cycle: twice per day (8 AM- 8PM); nose: 45 dB) and fed a standard laboratory pellet diet and water ad libitum.

(3) Please include the method of euthanasia & (4) Please describe the post-operative care received by the animals, including the frequency of monitoring and the criteria used to assess animal health and well-being;

To address this reviewer’s comment, we added some sentences in the method section as below :

At the end of this study, the animals were euthanized, according to the American Veterinary Medical Association guidelines for the euthanasia of animals. Briefly, under the deep anesthesia, the animals received bolus injections of potassium chloride.

3.Thank you for stating the following financial disclosure:

[The funders had no role in study design, data collection and analysis, decision to publish, or preparation of the manuscript.].

At this time, please address the following queries:

Please clarify the sources of funding (financial or material support) for your study. List the grants or organizations that supported your study, including funding received from your institution. State what role the funders took in the study. If the funders had no role in your study, please state: “The funders had no role in study design, data collection and analysis, decision to publish, or preparation of the manuscript.” If any authors received a salary from any of your funders, please state which authors and which funders. If you did not receive any funding for this study, please state: “The authors received no specific funding for this work.”

A) Following the conclusion, we inserted the sentence “The authors received no specific funding for this work”

Please include your amended statements within your cover letter; we will change the online submission form on your behalf.

A) The amended statements included within the cover letter

4. Please amend your manuscript to include your abstract after the title page.

A) Abstract is included

5. Review Comments to the Author

Please use the space provided to explain your answers to the questions above. You may also include additional comments for the author, including concerns about dual publication, research ethics, or publication ethics. (Please upload your review as an attachment if it exceeds 20,000 characters)

Reviewer #1: Kim et al. have reported that an intentional partial venous occlusion in the common iliac vein (CIV) of porcine with acute hindlimb ischemia created by embolization of the ipsilateral common iliac artery (CIA) increased the venous oxygen saturation. The report is interesting; however, there are several issues to be resolved for the next review.

1. There is no abstract in this manuscript.

A) Abstract is included

2. Although the CIA occlusion remained for 65 minutes in total, why did not the arterial oxygen saturation in the ipsilateral superficial femoral artery (SFA) decrease?

A) To address this reviewer’s comment, we added some explanations and modified the previous paragraphs in the discussion section as below :

Meanwhile, previous studies with the porcine hindlimb ischemic model have been limited by rapid collateralization [21,22]. Surgical CIA ligation would completely exclude the possibility of collateralization to the ischemic hindlimb and make a more durable and reproducible ischemic state in porcine model [20]. However, human peripheral limb vascular system has numerous collaterals and inter-individual disparities. Generally, irreversible tissue damage in acute limb ischemia usually occurs within 6 hours after complete arterial occlusion. Although endovascular CIA embolization lead to a stronger reduction in the total amount of blood flow into the ipsilateral limb, it would seem barely feasible to occlude all collateral branches at the initial stage of acute limb ischemia. Tissue blood flow and perfusion to the skin or limb muscles are significantly decreased, whereas intraluminal arterial saturation in the occluded artery may not be decreased and still has highly saturated blood. Abrupt total occlusion of the CIA, like surgical CIA ligation, is a rare medical scenario and is hardly seen in PAD patients clinically. Considering the mentioned points, our model is likely to show ischemic changes similar to those seen in patients with ALI. We focused on a straight-forward reliable and reproducible endovascular method. Moreover, our method is well equipped for performing our study which is designed to investigate the effect of partial venous occlusion on the oxygenation in porcine hindlimb ischemia.

3. The authors hypothesized that the intentional partial occlusion of the CIV congested the whole vein, subsequently increased the oxygen consumption in the ischemic limb tissue, and thereby increased the venous oxygen saturation. However, I cannot understand the hypothesis because an increase in the oxygen consumption in the ischemic tissue should further decrease the venous oxygen saturation. Could you clear the doubts away?

A) To address the reviewer comments and support our hypothesis, we add a paragraph in the discussion as below :

Enriched oxygen in tissue is considered beneficial to the metabolism in the muscle. The obstruction of arterial blood flow causes impaired oxygen delivery, tissue hypoxia and leads to the dysfunction of the electron transport chain in mitochondria. That induces anaerobic metabolism in cells, and the retention of lactic acid may lead to metabolic acidosis. Because tissue oxygen extraction/utilization is impaired in the ischemic limb, an increase in oxygen consumption in the ischemic tissue might not decrease the venous oxygen saturation [12]. Partial venous occlusion in the CIV induces local stasis of highly oxygenated blood in the ischemic area. It causes a slight venous pressure elevation in the vein, and the sustaining high oxygen level would be helpful for oxygen uptake by ischemic limb tissue, restoring cell metabolism and limb perfusion.

A) Figure 5 regarding venous pressure was added in the method (1), result (2) as below :

(1) Mean venous pressure were measured during the study period. All the mean venous pressure values were calculated over the stable period.

(2). Fig 5 shows a representative image of the mean venous blood pressure response during the study period. A significant decline after arterial embolization (from 18.9 ± 1.3 at T0 to 3.0 ± 1.2 mmHg at T1, p-value <0.001) and an obvious elevation were observed (from 3.0 ± 1.2 at T0 to 7.1 ± 1.3 mmHg at T1, p-value <0.001) in the mean venous pressure.

4. The authors showed an increase in venous oxygen saturation after the intentional partial occlusion of the CIV. However, the authors should additionally test whether the CIV occlusion could increase arterial oxygen saturation and tissue oxygenation in the peripheral arteries of the occluded CIA.

A) Arterial oxygen saturation was not changed significantly during the study. We mentioned about the aformentioned question, and inserted the explanation in the discussion as below:

Although endovascular CIA embolization lead to a stronger reduction in the total amount of blood flow into the ipsilateral limb, it would seem barely feasible to occlude all collateral branches at the initial stage of acute limb ischemia. Tissue blood flow and perfusion to skin or limb muscles are significantly decreased, whereas intraluminal arterial saturation in the occluded artery may not be decreased and still has highly saturated blood.

5. Please show if the intentional partial occlusion of the CIV can reduce ischemic damage (e.g. necrosis, inflammatory, oxidative stress, and apoptosis) in the ischemic limb.

A) We added a sentence in the result :

We did not detect a significant change in the level of carbon dioxide, bicarbonate or pH during the study period.

A) We added a response in the limitation :

Finally, our study was insufficient to demonstrate an effect of partial venous occlusion on the inflammatory marker, oxidative stress, and acid-base balance in the ischemic limb. However, this study was not designed or powered to look at the impact on those parameters, although those are likely of great interest. The study period may not have been long to detect significant changes.

6. The authors should additionally test the effect of the intentional partial occlusion of the CIV in porcine with chronic hindlimb ischemia, which is created by the CIA occlusion for several days. If impossible, the authors should not describe a clinical perspective regarding the CIV occlusion method for treating patients with critical limb ischemia.

A) Description regarding CLI were eliminated or modified in the introduction and discussion:

.The sentence ”Particularly in patients with CLI” was eliminated in the discussion.

.The whole paragraph was modified as below :

From “There are two fatal subgroups for PAD, critical limb ischemia (CLI) and acute limb ischemia (ALI). CLI is the most severe manifestation of PAD, being defined by ischemic rest pain, ulcers and/or gangrene and is usually caused by atherosclerosis in the lower extremity arteries. In contrast, ALI occurs when there is sudden decrease in limb perfusion that threatens limb viability and requires urgent treatment to prevent loss of limb [2]. Irrespective of the pathophysiological processes underlying the ischemia, the final results remain the same.” To “In particular, acute limb ischemia (ALI) occurs when there is sudden decrease in limb perfusion that threatens limb viability and requires urgent treatment to prevent loss of limb [2]. A lack of oxygen due to an interruption of the blood supply to an acutely occluded limb causes accumulation of various metabolites, production of reactive oxygen species (ROS) and an inflammatory reaction in conjugation with tissue swelling, threatening the possibility of limb loss and even death”

7. As for the statistical analysis of changes in the oxygen saturation following the intentional partial occlusion of the CIV, was the analysis adjusted by the baseline oxygen saturation before the occlusion?

A) adjusted

Reviewer #2: The authors present a single institution study looking at the effect of intentional restriction of venous return on tissue oxygenation in a porcine model of acute limb ischemia and investigate whether partial venous occlusion in the common iliac vein (CIV) causes a further increase of venous oxygenation in a porcine model of acute hindlimb ischemia.

1.Just under 10 porcines were analyzed. While this is interesting work, I have some questions and suggestions for revisions. **I think the number of the study is to small** to draw conclusion, **the authors should soften their statements**

A) To address this reviewer’s comment, we added some sentences in the discussion section as below :

Second, there are concern about the small number of experimental animals and a relatively short period of observation time. With the current amount of observations, however, there was obvious trend towards improvement of venous oxygen saturation over time, following intentional partial venous occlusion in the CIV. Possibly, the inclusion of an additional time point, e.g. 6 hours after the partial venous occlusion, may have revealed more information about the effect of intentional restriction of venous return on tissue oxygenation in the ischemic limb.

A) To soften the conclusion, final conclusion was modified as below :

Our results suggest potential positive therapeutic effect of partial venous occlusion to induce the enhancement of oxygen uptake in the ischemic limb tissue. The effect of partial venous occlusion on oxygenation in the ischemic limb may deserve to be investigated in patients for whom revascularization therapy is unsuccessful.

2. In Discussion part line 15 “An expectation for this may be that the oxygen consumption in the limb tissue is increased” do you mean expectation or explanation. I think you mean explanation.

A) Corrected (expectation->explanation)

3. The pathophysiological reasons of venous occlusion and increased oxygen saturation is not becoming clear. Please discuss it better. How do you explain this in the peripheral system?

A) To address the comment, we added a paragraph in the discussion, with adding the New figure 5 :

Partial venous occlusion in the CIV induces local stasis of highly oxygenated blood in the ischemic area. It causes a slight venous pressure elevation in the vein, and the sustaining high oxygen level would be helpful for oxygen uptake by ischemic limb tissue, restoration of cell metabolism and limb perfusion (Fig 5).

4.We know in the peripheral system a venous congestion results in trophic skin disorder and venus ulcera and is contraindicated for wound healing.

A) To address the reviewer’s comment, some explanation were inserted in the discussion as below :

On the other hand, there is concern that venous ulcer or venous stasis related life threating condition may occur due to venous congestion via the intentional partial venous occlusion causing pressure in the veins to increase. However, those catastrophic events are unlikely to develop because the intentional partial venous occlusion would be applied, temporarily.

5.How do you measure the saturation, this should be clarified.

A) A paragraph regarding gas analysis was inserted in the method as below :

Blood gas analysis was performed to measure oxygen saturation, carbon dioxide, bicarbonate, and acid-base balance. A proper blood sample for blood gas analysis consists of a 2 to 3 ml blood specimen collected anaerobically in 3 ml plastic syringe fitted with a small bore needle. Heparin was added to the syringe as an anticoagulant. Any air bubbles inadvertently introduced during sampling was promptly evacuated.

6. Page 3 line 21-23 : The authors try to transferr the pathophysiological process in the heart to the peripheral vessels. I think this need to be discussed much deeper. Arterial occlusion combined with venous occlusion results in the peripheral system to the situation phlegmasia coerlia dolens, which is a life threating condition. Who was lactate, do you measure any labor parameters.

A) Some paragraph supporting our hypothesis and answering the reviewer’s comment were inserted as below :

There is concern that venous ulcer or venous stasis related life threating condition may occur due to venous congestion via the intentional partial venous occlusion causing pressure in the veins to increase. However, those catastrophic events are unlikely to develop because the intentional partial venous occlusion would be applied, temporarily.

7. PLOS authors have the option to publish the peer review history of their article (what does this mean?). If published, this will include your full peer review and any attached files. If you choose “no”, your identity will remain anonymous but your review may still be made public. [NOTE: If reviewer comments were submitted as an attachment file, they will be attached to this email and accessible via the submission site. Please log into your account, locate the manuscript record, and check for the action link "View Attachments". If this link does not appear, there are no attachment files.]

While revising your submission, please upload your figure files to the Preflight Analysis and Conversion Engine (PACE) digital diagnostic tool, https://pacev2.apexcovantage.com/. PACE helps ensure that figures meet PLOS requirements. To use PACE, you must first register as a user. Registration is free. Then, login and navigate to the UPLOAD tab, where you will find detailed instructions on how to use the tool. If you encounter any issues or have any questions when using PACE, please email PLOS at figures@plos.org. Please note that Supporting Information files do not need this step.
