# Supplementary material for: Effect of intentional restriction of venous return on tissue oxygenation in a porcine model of acute limb ischemia
Source: PLoS One. 2020 Dec 14;15(12):e0243033. doi: 10.1371/journal.pone.0243033 (PMC7735909; doi:10.1371/journal.pone.0243033)
Supplement: Attachment — Submitted filename: Response to Reviewers (CIV occlusion study) 2nd revision.doc [file pone.0243033.s006.doc]

**Review Comments to the Author**

**Reviewer #1:** First of all, the authors’ response comments and explanations for the reviewer’s questions have been unkind for the reviewer. In order to accept the author’s explanation for the second comment of the reviewer, **it will be necessary some experiment to show numerous collaterals in the porcine limbs with acute ischemia.** If impossible, the authors should describe the concern in the section of Discussion. Next, according to the authors’ seventh response, **it seems that the authors have (had already?) performed some statistical analyses adjusted by the baseline data for the second (first?)** submission of this paper. The authors should describe that in the section of Materials and Methods:

**Prior to describe my answers, I would like to apologize for my unkindness in the previous answer letter (1^st^ revision rebuttal) I've caused. There are two comments raised by the academic editor and reviewer.**

**1^st^ comment answer :**

The background in PAD is extremely heterogenous with many factors involved, but the outstanding factor that limits the extent of ischemic limb damage following arterial occlusion is the degree of development of the collateral circulation. That is to say, development of collateral vasculature is key in compensating for arterial occlusion in the ischemic limb. Arterial anatomy of the porcine hind limb is different from that in human peripheral limb. We described (1) the different vascular collateral system in the pig, compared to that in human, and (2) the reason why the pig is likely to have a numerous collateral vessels in the setting of ALI model, as below :

In addition, arterial anatomy of the porcine hind limb is different from that in human peripheral limb. In the porcine vascular system, the distal abdominal aorta generally trifurcates into the right and left iliac arteries, and the common internal iliac trunk which then bifurcates into the right and left internal iliac arteries [24]. Development of collateral vasculature via those internal iliac arteries might be one of the important mechanisms in compensating for arterial occlusion in the porcine limb ischemic model. Our model was induced with arterial embolization in the CIA, not at the level of distal abdominal aorta. Thus, the patent common internal iliac trunk might facilitate the development of collateral blood flow into the ischemic limb.

From the reference 24 (Gao Y, Aravind S, Patel NS, Fuglestad MA, Ungar JS, Mietus CJ, et al. Collateral Development and Arteriogenesis in Hindlimbs of Swine After Ligation of Arterial Inflow. J Surg Res 2020;249:168-79. https://doi: 10.1016/j.jss.2019.12.005. PMID: 31986359).

**2^nd^ comment answer)**

We have only 7 experimental pigs in this study, and there are 4 estimated variables (T0, T1, T2, T3) prior to T4 (the time just prior to the occlusion of venous occlusion). Therefore, we confess that adjustment of those data is difficult in my statistical ability. Nevertheless, all pigs have a very similar baseline characteristics from the beginning of the experiment, including their size, weight, height, and a baseline arterial (T0 in artery; mean value of 100% O2 saturation without any standard deviation) and venous oxygenation (T0 in vein; mean value of 85.57 ± 1.72 % O2 saturation, just-small 1.72 standard deviation). We believe that our data is a relatively homogenous. Instead, to intensify the statistical significance of our result and compensate the un-adjusted data, we made a new paragraph and figure regarding the relationship between hypoxic damage by arterial embolization (defined as T4 minus T0 in vein saturation) and treatment effect with venous occlusion (defined as T7 minus T4 in vein saturation) by performing a Spearman correlation as below:

The sentence “Hypoxic damage by arterial embolization was defined as T4 minus T0 in vein saturation, and treatment effect with partial venous occlusion was defined as T7 minus T4 in vein saturation, respectively” was inserted in the method section.

A new figure (Fig 6) was created to compensate the unadjusted data in this study, strengthened the statistical power, and help readers to understand the study’s result more easily :

The sentence “Figure 6 shows that hypoxic damage by arterial embolization is positively associated with treatment effect with venous partial occlusion (r = 0.788, P-value=0.035). This strong positive correlation might be interpreted that the more severe hypoxic damage, the greater would be the treatment effect with partial venous occlusion.” was added in the discussion.

Fig 6. Spearman correlation was performed for bivariate correlations between hypoxic damage by arterial embolization (defined as T4 minus T0 in vein saturation) and treatment effect with partial venous occlusion (defined as T7 minus T4 in vein saturation) to establish a relationship.

**Reviewer #2:** No comments, the authors have adequately addressed my comments raised in a previous round.
