# Supplementary material for: Effect of intentional restriction of venous return on tissue oxygenation in a porcine model of acute limb ischemia
Source: PLoS One. 2020 Dec 14;15(12):e0243033. doi: 10.1371/journal.pone.0243033 (PMC7735909; doi:10.1371/journal.pone.0243033)
Supplement: Attachment — Submitted filename: Response to Reviewers (CIV occlusion study) 3rd revision.doc [file pone.0243033.s007.doc]

**Review Comments to the Author**

Reviewer #1: Thank you for your second response to my first review comments. I have gotten two answer comments and a revised manuscript based on the answer comments from you; however, there are two problems to be resolved in the revised manuscript.

**1)** If the authors cannot perform some experiments to show numerous collaterals in the porcine limbs with acute ischemia, the authors should describe that in the section of Discussion in the revised manuscript as follows: “...might facilitate the development of collateral blood flow into the ischemic limbs. However, we did not actually show the collateral arteries by some experiments, such as angiography and histological staining of the ischemic tissues, in this study.”

Answer) We thank you for your precious comments of our experiment. We added and modified some sentences to reflect your opinions as follows :

However, we did not actually show the limb perfusion through collaterals, which could be assessed with peripheral angiography, non-invasive imaging modalities, or histological staining of the ischemic tissues. Swine models fulfilling all ALI characteristics, with evaluating the adequacy of limb perfusion through collaterals intraprocedurally, seem to be lacking in the scientific literature. When it comes to further researches following this study, a major challenge may be having technologies available that can show numerous collaterals in the ischemic limb.

**2)** As to the second answer comment about the statistical analyses, it is necessary to be checked by a statistician whether the analyses were correctly performed to show the significance of their experimental results.

**Answer)** Actually, we consulted with a statistical specialist, when we prepared for the proper and best answers of the review’s previous comments. He (Dr. Byoung Geol Choi, PhD in Korea University Guro hospital, e-mail address: [trv940@naver.com](mailto:trv940@naver.com)) suggested one statistical technique-Pearson correlation coefficient, instead of statistical adjustment, because of a relatively small sample number in our study. At the present time, no one has been addressed our topic-partial venous occlusion in the ischemic limb.

If we were to perform statistical adjustment with our data, two methods-multiple linear regression or ANCOVA might be used. We (with Dr Choi) did those two methods (but, it didn’t work well). Calculating a sufficient sample size in number, based on our study’s result, we need at least 30 pigs to be sacrificed more (the minimal number for statistical adjustment, including pig’s death rate during the procedure). Even in the published researches regarding coronary sinus reduction in swine (the study which motivated us to start our topic), as we know, experimental pigs were small in number. And, all pig have a similar baseline characteristics, including age, sex weight, and nutrition, etc. We fully understand what the reviewer recommends. On the other hand, this is the first pilot study in literature (there is no previous or any article on this topic). At first, we just wanted to observe if partial venous occlusion in the common iliac vein (CIV) might cause a further increase of venous oxygenation. In the next study, we can make a special venous occluder to be deployed in vivo and have a plan to do an animal study with a large number of pigs, enough to do any statistical methods.

For all statistical methods used in our study, we consulted with Dr Byoung Geol Choi, PhD in Korea University Guro hospital. He have been participating in numerous projects, and published many clinical journals (e-mail address: [trv940@naver.com](mailto:trv940@naver.com)). In our data, Pearson correlation coefficient is the most appropriate statistical method that he recommended. We will try to do any statistical analysis, if the reviewer suggests a specific statistical methods for adjustment.

Compare 2 Means: 2-Sample Non-Inferiority or Superiority (Sample size calculation) with the result of our study.

The pig’s death rate (10-20%) during the experiment
